# Supplementary material for: Data-Driven Models Reveal Mutant Cell Behaviors Important for Myxobacterial Aggregation
Source: mSystems. 2020 Jul 14;5(4):e00518-20. doi: 10.1128/mSystems.00518-20 (PMC7363006; doi:10.1128/mSystems.00518-20)
Supplement: FIG S1 [file mSystems.00518-20-sf001.pdf]

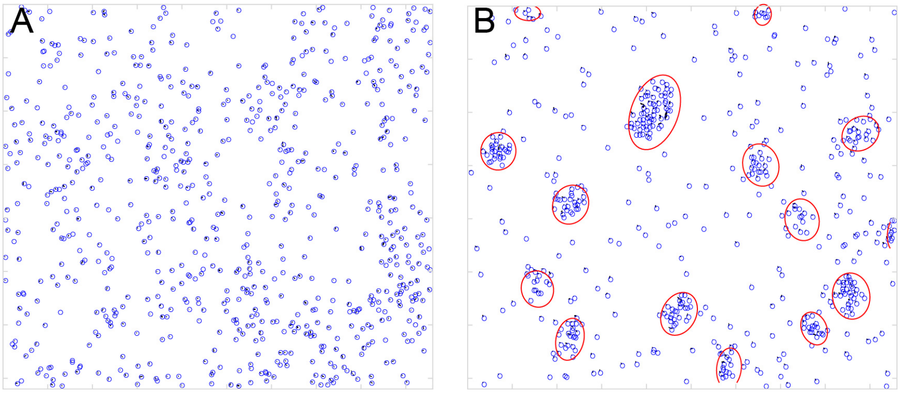

FIG S1 Aggregation result of WT cells from ref. (14). (A): Beginning frame of WT experiment. (B): Ending frame of WT experiment. Blue circles are labeled cells, red circles are aggregates.
