# Supplementary material for: Data-Driven Models Reveal Mutant Cell Behaviors Important for Myxobacterial Aggregation
Source: mSystems. 2020 Jul 14;5(4):e00518-20. doi: 10.1128/mSystems.00518-20 (PMC7363006; doi:10.1128/mSystems.00518-20)
Supplement: FIG S2 [file mSystems.00518-20-sf002.pdf]

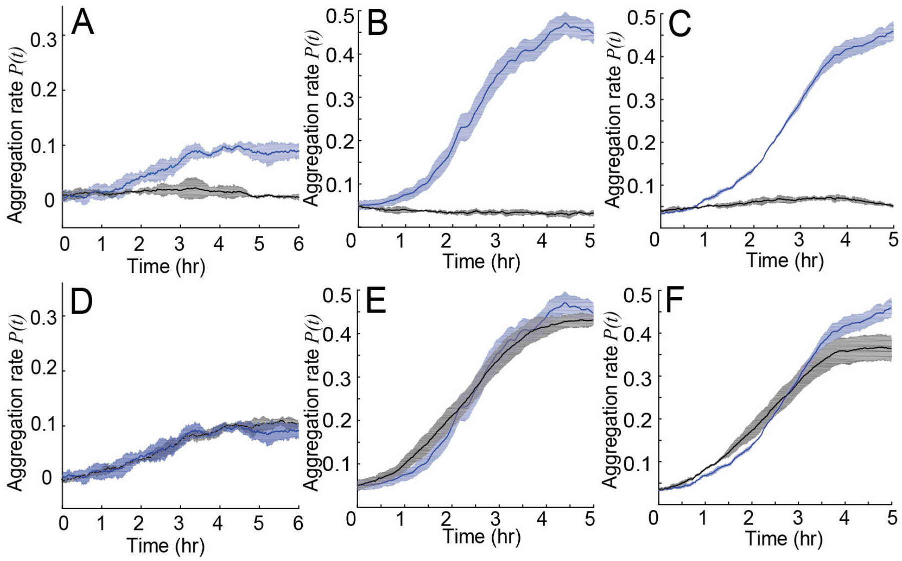

FIG S2 **Identification of key cell behaviors that drive mutant strain aggregation. Simulation results of *pilC* (A,D), *csgA* (B,E) and WT (C,F) based on the experimental data (quantified as $P(t)$, Eq. 1) on y-axis). Blue line and shaded areas are the simulation results under normal conditions. Black lines represent simulations without any dependence, i.e., data is randomly chosen (A-C) or simulation where run duration does not depend on time. (D-F). Shaded areas show standard deviations.**
