# Supplementary figures and images for: Data-Driven Models Reveal Mutant Cell Behaviors Important for Myxobacterial Aggregation
Source: mSystems. 2020 Jul 14;5(4):e00518-20. doi: 10.1128/mSystems.00518-20 (PMC7363006; doi:10.1128/mSystems.00518-20)

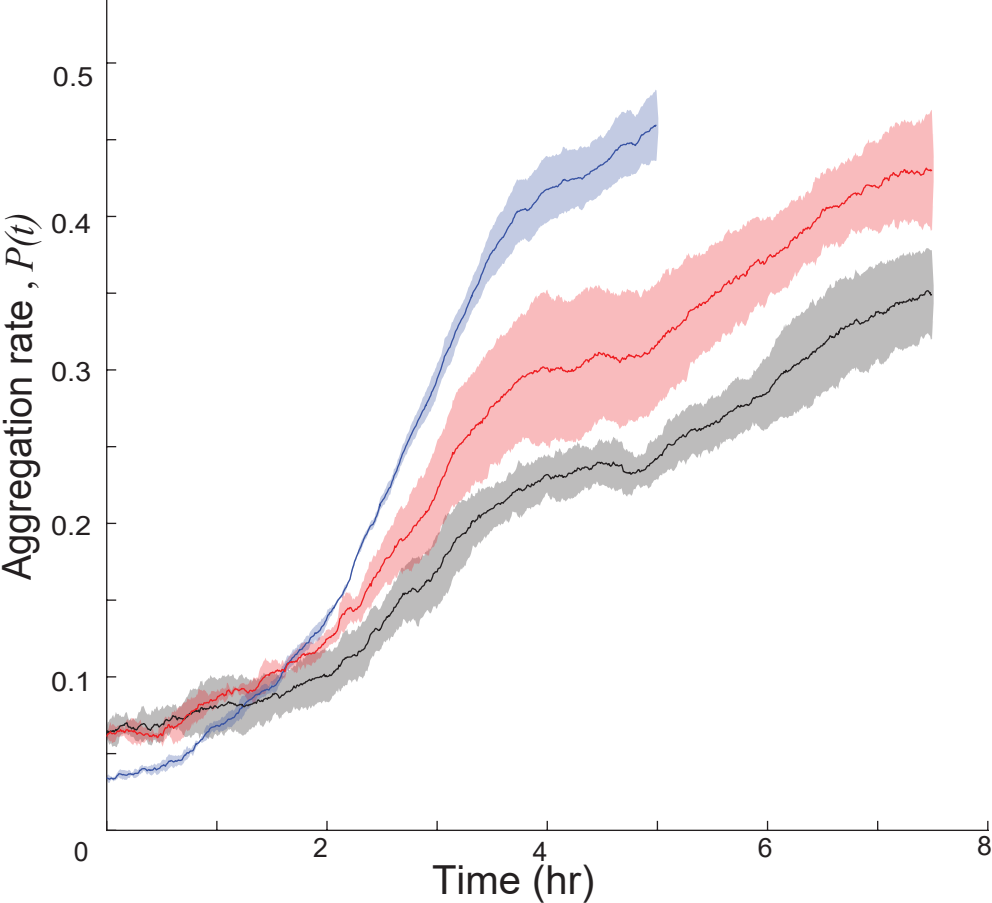

Supplement: FIG S3 [file mSystems.00518-20-sf003.pdf]

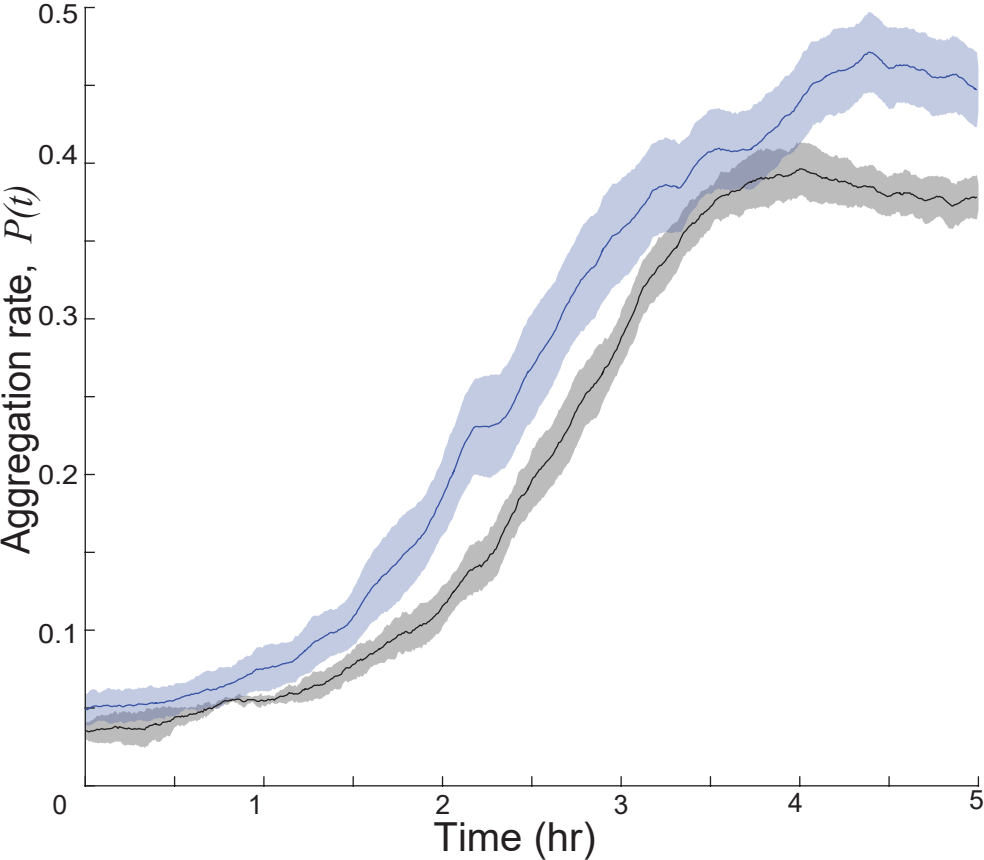

Supplement: FIG S4 [file mSystems.00518-20-sf004.pdf]

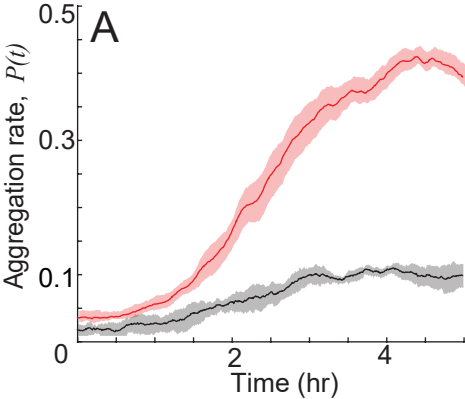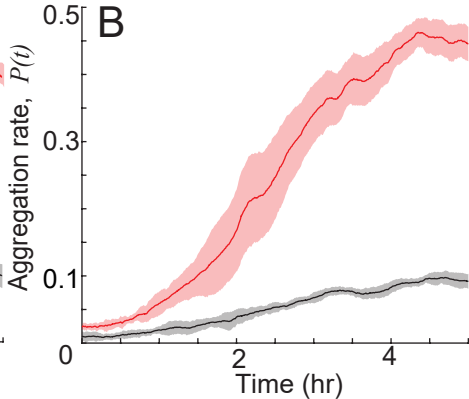

Supplement: FIG S5 [file mSystems.00518-20-sf005.pdf]

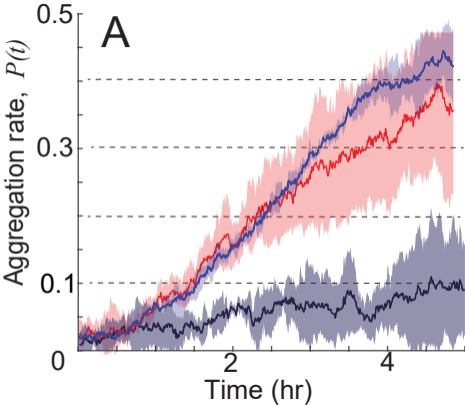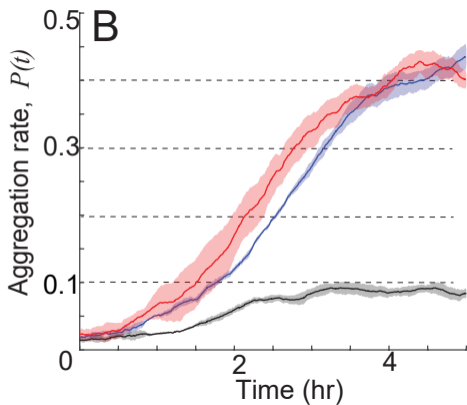

Supplement: FIG S6 [file mSystems.00518-20-sf006.pdf]
